# Acute effects of different balance exercise types on selected measures of physical fitness in youth female volleyball players

**DOI:** 10.1186/s13102-021-00249-5

**Published:** 2021-03-20

**Authors:** Raouf Hammami, Helmi Chaabene, Fatma Kharrat, Hanen Werfelli, Michael Duncan, Haithem Rebai, Urs Granacher

**Affiliations:** 1 Higher Institute of Sport and Physical Education of Ksar-Said, University of La Manouba, Tunis, Tunisia; 2 Research Laboratory Education, Motricity, Sport and Health LR19JS01, Higher Institute of Sport and Physical Education of Ksar-Said, Tunis, Tunisia; 3 Division of Training and Movement Sciences, University of Potsdam, Potsdam, Germany; 4 Research Laboratory: Education, Motricity, Sports, and Health, University of Sfax, Sfax, Tunisia; 5 Centre for Sport, Exercise and Life Sciences, Coventry University, Coventry, UK

**Keywords:** Postural stability, Conditioning activity, Short‐term effect, Team sports, Youth

## Abstract

**Background:**

Earlier studies have shown that balance training (BT) has the potential to induce performance enhancements in selected components of physical fitness (i.e., balance, muscle strength, power, speed). While there is ample evidence on the long-term effects of BT on components of physical fitness in youth, less is known on the short-term or acute effects of single BT sessions on selected measures of physical fitness.

**Objective:**

To examine the acute effects of different balance exercise types on balance, change-of-direction (CoD) speed, and jump performance in youth female volleyball players.

**Methods:**

Eleven female players aged 14 years participated in this study. Three types of balance exercises (i.e., anterior, posterolateral, rotational type) were conducted in randomized order. For each exercise, 3 sets including 5 repetitions were performed. Before and after the performance of the balance exercises, participants were tested for their static balance (center of pressure surface area [CoP SA] and velocity [CoP V]) on foam and firm surfaces, CoD speed (T-Half test), and vertical jump height (countermovement jump [CMJ] height). A 3 (condition: anterior, mediolateral, rotational balance exercise type) × 2 (time: pre, post) analysis of variance was computed with repeated measures on time.

**Results:**

Findings showed no significant condition × time interactions for all outcome measures (*p* > 0.05). However, there were small main effects of time for CoP SA on firm and foam surfaces (both d = 0.38; all *p* < 0.05) with no effect for CoP V on both surface conditions (*p* > 0.05). For CoD speed, findings showed a large main effect of time (d = 0.91; *p* < 0.001). However, for CMJ height, no main effect of time was observed (*p* > 0.05).

**Conclusions:**

Overall, our results indicated small-to-large changes in balance and CoD speed performances but not in CMJ height in youth female volleyball players, regardless of the balance exercise type. Accordingly, it is recommended to regularly integrate balance exercises before the performance of sport-specific training to optimize performance development in youth female volleyball players.

**Trial registration:**

This study does not report results related to health care interventions using human participants and therefore it was not prospectively registered.

## Background

In volleyball, dynamic balance, multidirectional locomotion, and jump-landing tasks such as spiking (offensive) and blocking (defensive), are key for performance [1, 2]. Previous studies have shown that balance training (BT) has the potential to induce performance enhancements in selected components of physical fitness (i.e., balance, muscle strength, power, [change-of-direction] speed) [3–5] and to increase resistance to injuries in children and adolescents [6–8]. Recently, Gebel et al. [9] summarized the effects of BT on selected components of physical fitness in the general youth population and youth athletes. These authors concluded that BT is highly effective to improve balance performance (e.g., static/dynamic steady-state) in youth (athletes). They further showed that BT has the potential to induce transfer effects to selected health- (e.g., muscle strength) and skill- (e.g., agility, speed) related components of physical fitness as well as on sport-specific skills (e.g., basketball shooting accuracy) in young athletes.

Additionally, there is evidence of facilitating effects from long-term balance training if conducted before strength or plyometric training [10–12]. For instance, Bruhn et al. [12] studied the sequencing effects of balance and strength training on maximum voluntary isometric contraction and neuromuscular activation in healthy physical education students aged 22 years. These authors demonstrated that a block of balance before strength training resulted in higher neuromuscular activation compared with strength followed by balance training. Accordingly, it was concluded that balance training has a facilitating effect on subsequent strength training. In other words, balance training contributes to optimize the effects of a subsequent strength training program.

Currently, there is no study available that examined whether the reported facilitating effects observed with long-term balance training also exist after a single balance training session. Prieske et al. [13] examined the acute effects of combined balance and strength exercises vs. strength exercises only on twitch contractile properties, maximum voluntary strength, and jump performance in young female soccer players. While the performance of strength exercises resulted in significant increases in twitch contractile properties, the combination of balance and strength exercises produced significant gains in jump performance. From these findings, it was concluded that neuromuscular adaptations such as intra- or intermuscular coordination rather than changes in contractile properties appear to be responsible for the observed jump height improvements following the performance of combined balance and strength exercises. Yet, no study has examined the acute effects of a single balance training session on physical fitness outcomes. If the performance of a single balance training session may also result in facilitating effects as was previously reported for long-term balance training, sport-specific training (e.g., volleyball training) should be conducted after a balance training session to optimize performance development.

In addition, it is unresolved whether the type of balance exercise (i.e., anterior, mediolateral, rotational type) moderates the performance outcome after the single balance training session. There is ample evidence that balance training leads to improvement in the trained but not the untrained task [14]. In a systematic review with meta-analysis, Kümmel et al. [15] demonstrated that balance training results in performance enhancements in the trained balance task with no-to-limited transfer to the untrained task. A recent study by Bakkum et al. [16] demonstrated that the more challenging the balance task, the larger the transfer effects to untrained movements. Taken together, this implies that balance exercises with larger degrees of freedom (e.g., rotational type) may have a greater effect on subsequent performance.

Therefore, this study aimed to examine the acute effects of different types of balance exercises (i.e., anterior, mediolateral, rotational type) on selected measures of physical fitness (i.e., balance, jump performance, and change-of-direction [CoD] speed) in female youth volleyball players. With reference to long-term balance training studies [11, 12], we expected a facilitating effect of a single balance exercise session on the above-mentioned key determinants of volleyball performance. Given that adaptations following balance training are highly task-specific [14, 15, 17] and appear to depend on the difficulty level of the respective balance task [16], we hypothesized that a balance exercise tool with larger degrees of freedom (i.e., rotational type) results in greater effects across the different outcome measures.

## Methods

A within-subject study design was applied to evaluate the acute effects of three different balance exercise types (i.e., anterior, mediolateral, and rotational type) on balance, jump performance, and CoD speed in youth female volleyball players. Participants were familiarized with the different physical fitness assessments (i.e., balance, CMJ, CoD) one week before the start of the study. Each session began with the players performing a standardized warm-up consisting of 5 min of jogging followed by 10 min of dynamic stretching. Thereafter, participants were tested (pre-assessment) for their balance, CoD speed, and CMJ height. Testing always started with balance tests followed by CoD speed and CMJ height tests. For the balance exercises, three different protocols (i.e., anterior, mediolateral, rotational type) were conducted in randomized order.

### Participants

With reference to the study of Prieske et al. [13] on the acute effects of balance and strength exercises on jump performance, an *a priori* power analysis with a type I error rate of 0.05 and 80 % statistical power was computed. The analysis indicated that overall, 10 participants are sufficient to observe significant, large-sized acute effects (Cohen’s d = 0.90) for countermovement jump height. Accordingly, this study was conducted with 11 youth female elite volleyball players, all recruited from the same club, who regularly competed on the national level (age: 14.3 ± 0.8 years, maturity offset [MO]: +0.9 ± 0.5, APHV: 13.4 ± 1.3 years, body mass: 56.9 ± 4.0 kg, body height: 172.2 ± 4.5 cm). All participants regularly played volleyball over the past 5 years before the start of the study. Throughout the study period, all athletes exercised 4–5 times per week with each session lasting ~ 90 minutes. The biological age of our participants was estimated using the maturity offset method [18]. It is worth noting that all subjects had regularly performed balance exercises as part of their regular conditioning program over the past 3 years. Thus, participants were familiarized with BT. This study was conducted in accordance with the latest version of the Declaration of Helsinki and the study procedures are in line with the standards for ethics in sport and exercise science research [19]. Before experimental testing, the study procedures were approved by the local institutional review board of the Higher Institute of Sports and Physical Education of Ksar Saïd, Tunisia. Written informed consent was obtained from parents/legal representatives of all participants before the commencement of the study.

### Procedures

All procedures were carried out during the second half of the competitive season (March-May 2019). Testing took place on three separate days at the same time of day (at 4 pm) and with 48 hours rest between days. One week before the commencement of the study, all subjects participated in an orientation session to become familiar with the testing procedure.

**Fig. 1:**
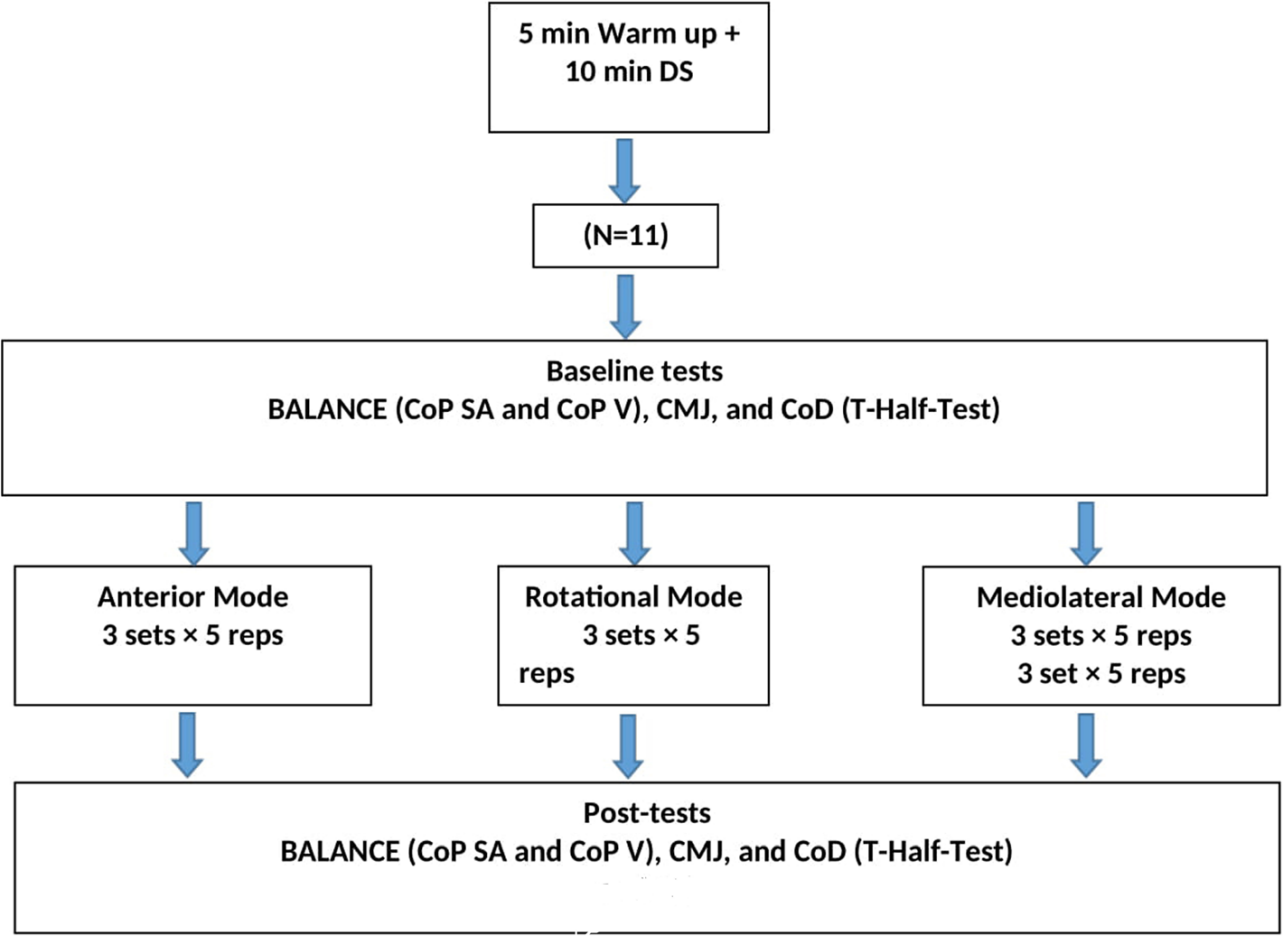
Experimental protocol of the three types of balance exercises.DS: Dynamic stretching, BT: balance training, CoP SA: center of pressure surface area, CoP V; center of pressure velocity, CMJ: countermovement jump height, CoD: change-of-direction speed

**Fig. 2:**
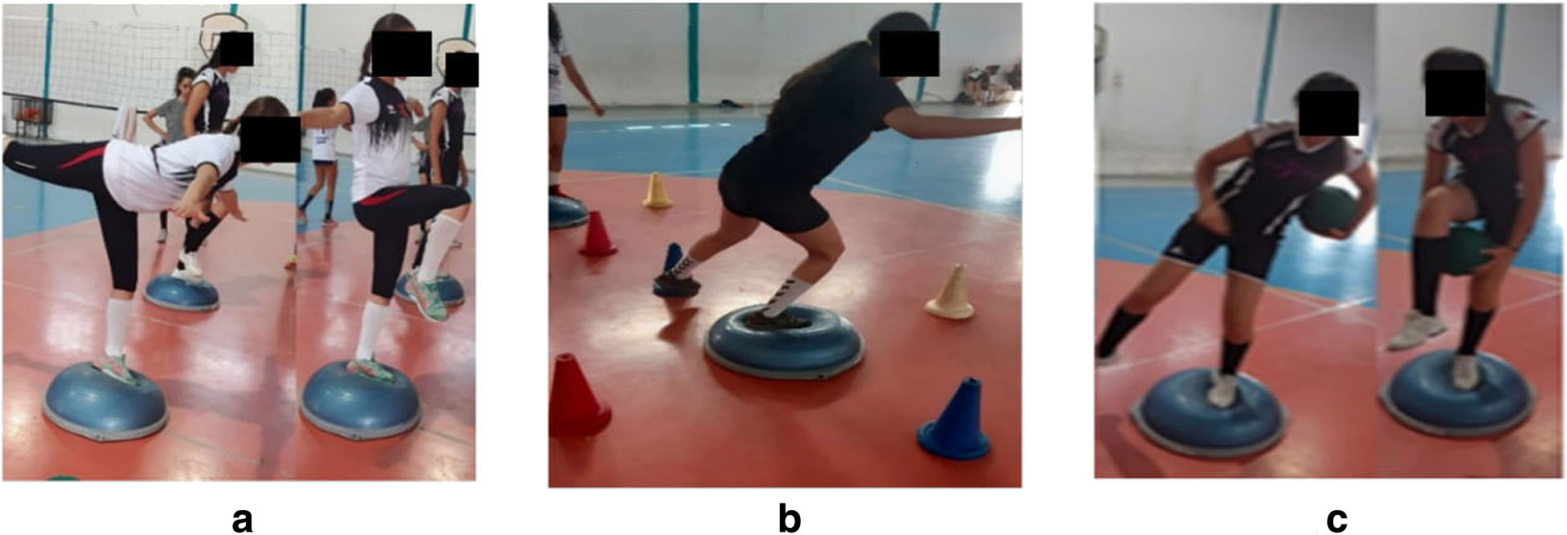
Schematic description of the three balance exercise types. **a **anterior balance exercise type, **b** mediolateral balance exercise type, and **c** rotational balance exercise type.

Each balance protocol lasted 15 minutes and consisted of performing single-leg stance exercises on unstable surfaces (i.e., BOSU). For each exercise, players completed 3 sets with 5 repetitions. Both legs were exercised (Fig. 1). A rest period of 90 seconds was allowed between sets. The anterior balance exercise protocol consisted of single-leg stance exercises with cutting motions of the contralateral leg in the sagittal plane (Fig. 2a). The mediolateral balance exercise protocol consisted of performing the same task while moving the contralateral leg laterally in the frontal plane (Fig. 2b). Finally, the rotational balance exercise protocol consisted of single-leg stance exercises while executing cutting motions of the hand and the contralateral leg around the body (Fig. 2c).

Participants were also instructed to refrain from any strenuous activities between the test sessions. To minimize confounding factors, instructions related to sleep and diet were given to all subjects before the experiment. On the night preceding each session, the subjects were asked to keep their usual sleep habits, with a minimum of 7 hours of sleep.

### Static balance

Static balance was evaluated using a force plate with three strain gauges (PostureWin©, Techno Concept ®, Cereste, France; 40 Hz frequency, 12-bits A/D conversion) which measures center of pressure displacements (CoP). The force plate was embedded in the surrounding floor. Participants were asked to stand as still as possible during testing with their arms comfortably placed downward at either side of the body, their bare feet were separated by an angle of 30° and their heels placed 5 cm apart. To maintain the same foot position for the balance assessment, a plastic device was used that allowed replication of the foot position. Participants were asked to maintain balance with eyes opened (EO) on firm and foam surfaces. Throughout testing, participants were instructed to look straight ahead at a cross, placed at eye level on a nearby wall (2 m distance). Each trial lasts 25.6 s. In this study, CoP sway parameters (i.e., CoP SA and CoP V) on foam and firm surfaces were analyzed. More specifically, CoP V indicates the total distances covered by the CoP divided by the duration of the sampled period and CoP SA represents the ellipse of the area covered by the trajectory of the CoP [20]. For these parameters, the lower the value, the better the postural control [21].

### Jump performance

CMJ height was evaluated using an Ergojump System (Ergojump; Globus Italia, Codogne, Italy) as was previously described [22]. During the performance of CMJs, participants were instructed to place their hands on the hips to prevent any support of arm movements on vertical jump performance. Participants were instructed to begin the jump with a downward movement, which was immediately followed by a concentric upward movement, resulting in a maximal vertical jump [23]. They were instructed to minimize lateral and horizontal displacements during performance. Proper care was taken to ensure correct technical execution (e.g., extended legs during flight-time). Three trials were performed with approximately 2 minutes rest in between trials. The best trial in terms of CMJ height was used for further analysis.

### Change‐of‐direction speed

CoD speed was evaluated using the T-half test as previously outlined by Haj Sassi et al. [24]. The T-Half test was used to determine speed with directional changes such as forward sprinting, left and right shuffling, and back pedaling. Participants began with both feet behind the starting line. At his discretion, each participant sprinted forward to the cone fixed at 5 meters and touch the base of it with the right hand. Facing forward and without crossing feet, they shuffled to the left to cone fixed at 2.5 meters and touch its base with the left hand. Subjects then shuffled to the right to the last cone and touch its base with the right hand. They shuffled back to the left to the first cone and touch its base. Finally, participants ran backward as quickly as possible and return to starting line. Any participants who crossed one foot in front of the other, failed to touch the base of the cone, and/or failed to face forward throughout had to repeat the maneuver. A total of three trials was carried out by each participant with 3 min of rest in-between. The best performance was recorded for further analysis.

### Statistical analyses

Data are presented as means and standard deviations (SDs). Data were tested and confirmed for normal distribution using the Shapiro-Wilk test. To evaluate the acute effects of the different balance exercise types on measures of physical fitness, a 3 (condition: anterior, mediolateral, rotational balance exercise type) × 2 (time: pre, post) analysis of variance (ANOVA) was computed with repeated measures on time. If condition × time interactions reached the level of significance, post-hoc tests (i.e., paired sample t-tests) were computed to identify the comparisons that were statistically significant. Effect sizes (ES) were determined by converting partial eta-squared from the ANOVA output to Cohen’s d. ES can be classified as trivial (< 0.2), small (0.2–0.49), medium (0.5–0.79) or large (≥ 0.8) [25]. Test-retest reliability was assessed using the intraclass correlation coefficient (ICC) and the standard error of measurement (SEM) expressed as coefficient of variation [26]. The alpha level of significance was set at p < 0.05. All data analyses were performed using SPSS 26.0 (SPSS, Inc., Chicago, IL, USA).

## Results

### Reliability analyses

No test or training-related injuries were observed throughout the study. Thus, the final data set comprised all initially enrolled eleven players. Table 1 illustrates performance data reliability outcomes for the applied physical fitness tests. Cronbach’s alpha intra-class coefficient correlations showed acceptable reliability with ICCs ranging between 0.82 and 0.91 (95 % CI: 0.84–0.96) and CVs ranging between 0.86 and 1.14.

**Table 1:** Test-retest reliability of the applied balance, jump, and change-of-direction speed tests

Criterion measures	ICC_3.1_ (95 % CI)	CV (%)
CoP SA Firm	0.88 [0.71–0.95]	2.75
CoP SA Foam	0.82 [0.75–0.87]	2.86
CoP V Firm	0.84 [0.80–0.87]	2.08
CoP V Foam	0.85 [0.81–0.88]	1.14
CMJ	0.91 [0.77–0.97]	2.44
CoD-Speed	0.91 [0.78–0.96]	2.80

Notes: Values are means and standard deviations (SD), *ICC *intra-class correlation coefficient, *CV* coefficient of variation, *CoP SA Firm* Center of pressure surface area on firm surface, *CoP SA Foam* Center of pressure surface area on foam surface, *CoP V Firm* Center of pressure velocity on firm surface, *CoP V* Foam Center of pressure velocity on firm surface, *CMJ* countermovement jump, *CoD* change of direction

### Static balance

Our findings indicated significant main effects of time for CoP SA on firm and foam surfaces (F_(1,30)=_4.22; d = 0.38; *p* < 0.05 and F_(1,30)_ = 4.30; d = 0.38; *p* < 0.05, respectively). For CoP V on firm and foam surfaces, no significant effects of time were observed (F_(1,30)_ = 1.78; d = 24; *p* > 0.05; F_(1,30)_ = 1.50; d = 0.22; *p* > 0.05, respectively). In addition, no significant condition × time interactions were found for all balance variables (F_(2,30)_ = 0.19–2.43 ; d = 0.11–0.40; *p* > 0.05) (Table 2).

**Table 2:** Acute effects of three different balance exercise types (i.e., anterior, mediolateral, rotational type) on measures of balance, jump performance, and change-of-direction speed in youth female volleyball players

	Anterior		Mediolateral		Rotational		ANOVA (*p* value; effect size)	
	**Pre**	**Post**	**Pre**	**Post**	**Pre**	**Post**		
	**M (SD)**	**M (SD)**	**M (SD)**	**M (SD)**	**M (SD)**	**M (SD)**	**Time**	**Condition x Time**
CoP SA Firm	107.2 (48.1)	97.4 (30.7)	107.3 (48.1)	140.6 (74.9)	107.3 (48.1)	141.4 (61.8)	**0.049 (0.75)**	0.105 (0.80)
CoP SA Foam	206.0 (89.8)	97.4 (29.3)	206.0 (89.8)	273.0 (150.3)	206.1 (89.8)	268.1 (121.8)	**0.047 (0.75)**	0.824 (0.22)
CoP V Firm	8.36 (1.0)	8.52 (1.4)	8.36 (1.0)	9.11 (1.4)	8.4 (1.0)	8.6 (1.6)	0.191 (0.45)	0.668 (0.33)
CoP V Foam	12.8 (1.7)	12.5 (1.5)	12.9 (1.7)	12.9 (4.0)	12.9 (1.8)	11.1 (1.5)	0.230 (0.44)	0.395 (0.45)
CMJ height	25.4 (2.7)	25.4 (3.5)	25.4 (2.7)	25.7 (3.5)	25.4 (2.7)	25.5 (3.4)	0.554 (0.22)	0.958 (0.10)
CoD speed	13.1 (0.371)	12.9 (0.477)	13.1 (0.371)	12.9 (0.55)	13.1 (0.371)	12.8 (0.502)	**< 0.001 (1.82)**	0.241 (0.62)

Notes: Values are expressed as means and standard deviations (SD), *CI* 95 % confidence limits, *CoP SA Firm* Center of pressure surface area on firm surface, *CoP SA Foam* Center of pressure surface area on foam surface, *CoP V Firm* Center of pressure velocity on firm surface, *CoP V Foam* Center of pressure velocity on foam surface, *ANOVA* analysis of variance, *Time* main effect of time (pre, post); Condition: anterior, mediolateral, rotational type

### Jump performance

Results showed no significant main effect of time (F_(1,30)_ = 0.36; d = 0.11; *p* > 0.05) for CMJ height. Likewise, no significant condition × time interactions (F_(2,30)_ = 0.04; d = 0.05; *p* > 0.05) were observed (Table 2).

### Change‐of‐direction speed

Our analysis showed a significant main effect of time (F_(1,30)_ = 24.84; d = 0.91; p < 0.001) but no significant condition × time interaction (F_(2,30)_ = 1.49; d = 0.09; p > 0.05) (Table 2).

## Discussion

This is the first study to investigate the acute effects of different types of balance exercises (anterior, mediolateral, and rotational type) on measures of balance, jump performance, and CoD speed in youth female volleyball players. The main findings indicated small-to-large acute changes in balance and CoD speed performances but not in jump performance, irrespective of the applied type of balance exercise.

Balance is an essential component of training in youth [3, 4, 27]. Earlier cross-sectional studies demonstrated that balance performance is associated with other measures of physical fitness such as jumping height and CoD speed performance in young athletes [28, 23]. Likewise, medium-to-large associations were reported between static (Stork balance test) and dynamic (Y-balance test) balance performances with proxies of muscle power (i.e., standing-long jump, countermovement jump) and CoD speed performances in male soccer players aged 10 to 16 years [23]. While there is compelling evidence from cross-sectional studies about the association of balance with measures of muscle power and CoD speed, only one study [13] examined the acute effects of combined balance and strength exercises vs. strength exercises only on twitch contractile properties, maximum voluntary contraction of the plantar flexors, and jump performance in young female soccer players aged between 14 and 15 years. Results indicated that the combination of balance and strength exercises significantly enhanced subsequent jump performance but not twitch contractile properties [13].

The current study showed that balance exercises only resulted in acute small balance performance improvements. Balance involves the interaction of automatic postural and voluntary motor commands of both the trunk and limb musculature [27, 29]. With reference to longitudinal studies [3, 30, 31] it seems plausible to argue that the general adaptive mechanisms that occur in the level of the spinal and supraspinal centers in the youth population after weeks of training (e.g., increased afferent feedback to cortical and/or subcortical areas, increased presynaptic inhibition) can be observed after a single bout of balance exercise. In other words, with reference to the aforementioned studies, it seems logical to argue that the similar acute improvement in balance performance following anterior, mediolateral, and rotational bout of balance exercises could be related to the acute physiological changes in spinal and supraspinal areas. However, these adaptations are transient and can only transform into persistent adaptations after long-term training [3, 30, 31]. Thus, coaches, as well as strength and conditioning professionals, should systematically implement balance exercises into warm-up to improve acute balance performance.

Further, irrespective of the type of balance exercise, our findings showed large effects on subsequent CoD speed performance. To the authors’ knowledge, this is the first study that examined the acute effects of different balance exercise types on CoD speed performance. The successful execution of the T-half test requires good capabilities to rapidly accelerate, decelerate, and change position from side-to-side. In such demanding situations, the balance system needs to compensate and adjust as the CoD speed task requires to repeatedly shift the center of gravity outside the base-of-support which challenges body equilibrium [17]. There is evidence from cross-sectional studies that shows medium-to-large-sized correlations between CoD speed and balance performance [23, 32]. In this context, it has been demonstrated that balance is an important prerequisite for efficient CoD speed performance [33]. The present study indicated that balance exercises seem to trigger acute mechanisms that contribute to better CoD speed performance in youth female volleyball players. However, further studies are required that examine the underlying mechanisms responsible for the observed changes in CoD speed performance after a bout of balance exercise.

This study revealed that the performance of balance exercises did not impact jump performance (i.e., CMJ height). To the authors’ knowledge, no previous study has examined the acute effects of different balance exercise types on CMJ height. Therefore, our findings have to be compared with studies examining the acute effects of different exercise types (i.e., whole-body vibration [WBV]) on jump performance. Of note, our results are in line with those of Kurt and colleagues [34] who showed that vertical jump performance was not affected by a single bout of WBV in well-trained combat athletes. In contrast, Cochrane and Stannard [35] assessed the acute effects of WBV on vertical jump performance and reported a positive effect on CMJ height. Furthermore, previous studies have shown that the greater the training experience and/or expertise level, the less likely it is to achieve large performance improvements [36, 37]. In this study, elite female volleyball players were included who regularly practiced volleyball for the last 5 years with 4–5 training sessions per week. Given that well-developed vertical jump height represents an important prerequisite for volleyball performance [1, 38], it can be argued that the recruited sample of female players was already close to the ceiling in terms of their jumping performance which again reduces the likelihood of achieving extra performance gains following a single balance training session. Of note, the acute effects of balance exercises are transient. In the present study, the CMJ height test was performed ⁓15 minutes after the balance exercise protocol. Therefore, it seems legitimate to argue that any potential effects of the applied balance exercise protocol may have mitigated over time. Therefore, further research is needed to substantiate the current findings.

This study is not without limitations. First, we are aware that our study findings are based on a relatively small sample. Given that we conducted this study with elite female youth athletes, the overall cohort (female youth athletes) to draw our sample from is small. Therefore, future studies should try to include a larger cohort of youth players – if available. Second, the lack of a control condition constitutes another limitation of this study. Therefore, future studies should replicate findings from this study and include a control condition. Third, systematic bias due to learning effects cannot be completely ruled out. However, the participating players were familiar with the applied test protocols (i.e., CoD speed and jumping) because they were frequently included in their training routines as part of performance testing. In addition, a familiarization session was scheduled one week before the start of the study. As such, potential learning effects cannot be completely ruled out but are most likely negligible and should not have biased the outcomes of this study. Finally, we were not able to examine the underlying neuromuscular mechanisms responsible for the observed changes in measures of physical fitness due to the lack of including neurophysiological testing apparatus in the design of this study. Therefore, future studies are advised to include electrophysiological testing apparatus (e.g., electromyography) to elucidate the underlying neural changes.

## Conclusions

The results of this study indicate that balance exercises in the anterior, mediolateral, and rotational planes produced small-to-large acute changes in balance and CoD speed but not in vertical jump height in female elite youth volleyball players. Coaches and strength and conditioning specialists are advised to regularly integrate balance exercises before the performance of sport-specific training to optimize performance development. Further research is needed to assess the exact balance exercise dosage required to stimulate improvements in jumping performance and whether our findings can be translated to different populations. 

## Data Availability

The datasets generated and/or analysed during the current study are not publicly available. Upon request, the corresponding author will share the data set.

## Abbreviations

BT: Balance training
CoD: Change-of-direction
CMJ: Countermovement jump
PHV: Peak height velocity
APHV: Years from peak height velocity
OE: Opened eyes
BM: Body mass
BMI: Body mass index
ICC: Intraclass correlation coefficient
CV: Coefficient of variation
CoP SA Firm: Center of pressure surface area on firm surface
CoP SA Foam: Center of pressure surface area on foam surface
CoP V Firm: Center of pressure velocity on firm surface
CoP V Foam: Center of pressure velocity on firm surface
CI: Confidence interval
M (SD): Means and standard deviations
ANOVA: Analysis of variance
